# Pennsylvania State Dental Society

**Published:** 1886-09

**Authors:** Wm. H. Trueman


### PENNSYLVANIA STATE DENTAL SOCIETY.

### EIGHTEENTH ANNUAL MEETING.

REPORTED FOR THE INDEPENDENT PRACTITIONER BY WM. H. TRUEMAN, D. D. S.

The Pennsylvania State Dental Society convened at the Mountain House, Cresson, on Tuesday, July 27, 1886, and was called to order by the President, Dr. J. W. Rhone, of Bellefonte.

The morning session was occupied by routine business.

At the opening of the afternoon session the President read his address, which was somewhat of a new departure. In place of the usual platitudes of such addresses, the doctor gave a brief and concise *resumé* of various matters of business originating or referred to at the last session, and requiring further attention at this. The good effect of his timely and judicious suggestions in the prompt and orderly transaction of business, was seen through the entire session.

Dr. E. H. Neal, of Philadelphia, read a paper entitled "A Review of the Causes of the Decline of Mechanical Dentistry." He remarked that, looking over the pages of our dental journals, one would be led to suppose that mechanical dentistry was fast becoming a thing of the past; indeed, so little has been written upon it, and so seldom is it referred to in our society meetings, that it seems to have ceased to be of special interest to many practicing dentists.

Quite recently, however, considerable interest has been manifested in it by the introduction of bridge-work. This he considered a step in the right direction, although it is, perhaps, too early to speak decidedly as to its merits. While there is much in it to commend, it is not without faults. It is frequently spoken of as unclean, opposed to sound mechanical principles, and it is contended that the severe strain imposed upon a few teeth will soon dislodge them.

He contrasted the dental laboratory of thirty years ago with that of to-day. Then its secrets were carefully guarded, and its recipes and processes either brought a high price or were communicated under a pledge of secrecy. Then the preservation of natural teeth received far less attention, and the insertion of substitutes was a much more prominent feature of dental practice than at the present time. The teeth were usually made to suit each case, the dentist not only carving the teeth he inserted, but also preparing the body, enamel, etc., from the crude materials. He exhibited a number of specimens of his own and his father's handiwork, all made more than thirty years ago, and all excellent specimens of that class of work, rivaling in appearance and expression the best that is being produced at the present time. He described the method of making the old-style riveted blocks, and thought that in skilled hands that now antiquated method was capable of better results in expression and usefulness than is usually obtained with moulded teeth. They had an individuality that is so often lacking in artificial dentures of more recent date. He referred to the time required to become a skilled carver of teeth, and the constant care required at each stage of the process; of the difficulties to be met and surmounted, and the constant liability to accidents requiring hours of labor to repair. He regretted that block carving was not more thoroughly taught in the dental colleges, as to produce satisfactory results so many cases require the teeth to be specially carved to suit them.

He suggested that the introduction of easier methods had developed a carelessness and indifference to the minuter details of constructing artificial dentures, and had in a measure passed that department of our specialty into the hands of those who did not appreciate the varied conditions to be met, but regardless of the anatomy of the mouth, its varied pathological conditions or the peculiarities of the patient, used teeth of the same mould, size and color, for all cases.

He then referred to the constantly increasing attention given to the preservation of the natural teeth. This has not only materially reduced the necessity for artificial substitutes, but has also, to a very great extent, withdrawn talent and skill from the laboratory, leaving that department in far inferior hands, the easier methods of the present having failed to develop the same skill as did the severe training of the past.

In conclusion, he suggested the following causes for the decline of mechanical dentistry:

First—The efforts of dentists, and the knowledge diffused by numerous publications upon the subject, have directed public attention to the importance of preserving the natural teeth.

Second—The vast improvements in material and implements have rendered operations looking to the preservation of the natural teeth more useful and reliable. For this the thanks of patients and operators are justly due to inventors and manufacturers who have so well supplied our needs.

Third—The ease and comparative freedom from pain with which the work is done.

DISCUSSION.

*Dr. Beck*—Referring to his early experience, spoke of the many accidents liable to occur in carving blocks, such as "gasing" the gums, that is, the gases given off from the fuel used in baking them passing into the muffle and acting upon the coloring matter of the enamel, changing its color to a dark blue or deep purple. When the blocks were made the difficulty was not over; riveting the blocks to the plate involved considerable risk. With the essayist, he thought the only proper way to construct an artificial denture was to make the teeth for the individual, carving and coloring them to suit each special case.

*Dr. J. C. Green*—Referred to Dr. Foster, of Trenton, N. J., the Wardell Brothers, Drs. Hall and Neal (father of the essayist), of Philadelphia, as men who had many years ago a reputation as skillful block carvers. No one at the present time can appreciate the difficulties dentists then labored under. Now, everything is prepared ready for use, and professional secrets are unknown; then, the dentist had to prepare everything himself, and to study out, unaided, as best he might, the many difficulties he encountered. He referred to Dr. Wildman's patient investigations, looking to the preparation of a reliable gum color, to his success, and to his giving to the profession so freely the results of his labors. He well remembered the relief it was to him when the improved gum color was introduced. With Dr. Beck, he thought that to have teeth look natural they must be carved for the individual.

*Dr. Magill*—Thought that some teeth made in Europe had a more natural and bony appearance than many made here.

*Dr. Templeton*—Referred to errors in arranging teeth that he had frequently noticed, especially that of allowing the molars to curve in, making the arch in shape somewhat like a horseshoe. Some dentists seem to have but one mould, inserting in nearly every mouth small white teeth, without the slightest regard to individual requirements.

During the discussion the question was raised whether there had been a practical decline in the art of constructing artificial dentures. It was freely admitted that less labor, and perhaps less skill, was now expended, and on the other hand contended that the general use of the atmospheric principle in place of clasps and the more cumbersome springs, the improvements in pivoting, etc., indicated a decided advance, and that the dentures of the present were worn with more satisfaction and comfort than were the productions of the past.

A paper upon Dental Anomalies, presented by Dr. L. Campbell, of Slatington, was read.

The doctor accepted Magitot's definition of anomaly—a deviation from a specific type—and gave to Magitot the credit of having been the first to collect and to arrange for convenient study such facts as were then known relating to Dental Anomalies. He stated it as conceded that all defects of organism originate in embryonic peculiarities, and admitting this, we have little difficulty in recognizing the cause of many peculiarities of the dental system.

He urged the importance of carefully noting and studying the history of the many abnormal conditions met with in practice. In course of years these records may prove of great scientific value. He related a number of cases of persistent deciduous teeth, of delayed dentition, and of abnormal cases where teeth missing from the arch had erupted long after the usual time, and, in some cases, far from the place they usually occupy.

### EVENING SESSION.

Dr. Louis Jack, of Philadelphia, read a paper entitled "Distinctions between the Indications of Hyperæsthesia, and those of Disturbances of the Blood Vessels of the Dental Pulp."

He said there are manifested as a consequence of the irritation set up in the tissues of the pulp, brought about by the encroachment of caries, two distinct series of symptoms, one pertaining to the nerves of the structure, the other pertaining to the state of the circulation of the pulp. These are also respectively called the subjective and objective indications. To obtain a correct understanding of these conditions, some consideration of well-known anatomical peculiarities of the organ is necessary.

First, in regard to the arrangement and ultimate distribution of the nerves. It has been clearly shown that nerves enter the foramen by several distinct fibers which pass in an upright and parallel direction, giving off but few branches, and anastamosing but little until they reach the bulbous portion of the organ, where they form an exceedingly rich plexus, situated immediately beneath the odontoblastic layer. Thus far, observers, if we accept the unsupported claims of Boll, have not discovered nervous filaments beyond the peculiar boundary of loops formed by the ultimate terminations of the capillaries. We have to await further investigation to determine whether the ultimate distribution is only to the last limit of the blood-vessels, or whether they pass between the odontoblastic processes, the remains of which, after calcification is complete, constitute the dental fibrils. The fact that the surface of the pulp is not a highly sensitive organ to simple touch, not accompanied by pressure, may indicate that the latter distribution of the terminals is not correct, and that Boll's observations were incomplete. The absence of sensation, however, is not absolute proof of the absence of nerves. Many of the deeper structures are devoid of sensibility, until the activity of the nerves of sensation are awakened by increased blood supply. But it is a fact of great interest in connection with the treatment of the pulp, that the organ is not quickly responsive to chemical irritation.

The afferent vessels of the dental pulp enter with the nerves, and pursue a similar direct course, breaking up by branching during their passage, and at length form a capiliary plexus over the surface of the organ. This capillary plexus has the remarkable characteristic that the ultimate capillaries do not anastamose with each other at the plane of their ultimate distribution, but that one side of the loop is afferent and the other efferent. This arrangement seems to be of the utmost significance, since the tendency of it is to limit the circulatory disturbance to the point of irritation, and is therefore restrictive of the diffusion of the excitement; it also tends to limit the nervous excitement of the organ, since exalted sensation and increased blood supply are correlative.

The situation of the pulp is such that it is subject to constant concussions, to thermal shocks, and, when decay commences, to chemical irritation sufficient, we would infer, to excite derangement of its functions; but so far is this from being the case that, in a great majority of instances of encroachment of caries upon the pulp, the cause of pain is not so much the altered relation or the chemical irritation, as the direct application of force by which fluids are pressed against the delicate tissues of which the organ is composed. With this begins what the doctor terms the subjective stage of pulp irritation, indicated by a series of symptoms, varying in intensity and character in different individuals, the variations being no doubt due to constitutional differences and the varying intensity of the irritation excited. With some there may be repeated attacks of neuralgic pain in some of the branches of either division of the trigemini; in others it may find expression as pain in some other tooth, or a long continued frontal headache, lasting for weeks and occurring at stated periods, or the pain may shift from day to day to any portion of the trigeminal sphere. These symptoms all have a tendency to recurrence in the evening, and after they become established constantly tend to evening attacks. This last becomes the salient means of distinguishing between a case of dental irritation and an attack of facial neuralgia due to malarial poison, which occurs at regular periods, but less frequently at night. The above does not continue long without the occurrence of a marked symptom, which invariably attends the subjective stage when the principal element involved is the nervous tissue of the organ. This is the responsiveness of the tooth to cold. This impressibility to reduced temperature is peripheral, and appears to extend throughout the whole of the enamel. It is so persistent during the subjective stage, and varies so greatly in degree, that it may be accepted as an index of the degree of the hyperæsthesia, and it may be employed as a test of the progressive recovery, or, on the other hand, of the progressive advancement toward a more serious condition.

After the subjective indications have continued for a variable time, a new series of symptoms may set in, and for a time are associated with the first, which have so far been of a reflected character.

This new phase of pulp disturbance is denominated the objective stage, and its symptoms are manifested in the tooth itself. They usually occur, when they do not arise as the direct consequence of decided compression or mechanical injury, in a sequential order. First, there is some sensation of the tooth to contact, and it responds when struck with an instrument as it did not in the previous stage. This is due to congestion of the peridental membrane. The reason for it is found in the fact that the peridental membrane, near the apex, derives a large portion of its circulation from the vessels as they approach the foramen; the nervous excitement induces a determination of blood which, being restricted from entering by the minuteness of the foramen, is partly distributed upon the membrane at the apex, and excites the sensibility of the nerves here, of which there is a free supply. Accompanying this condition is found an increased sensibility of the pulp, manifested at length by a heavy boring or a throbbing pain, caused by the increased determination of blood and the plethoric condition of the vessels of the pulp. This pain is continuous, but more severe at night. At last, as the congestive condition increases, the pulp is usually devitalized as a consequence of strangulation of the tissues at the point of entrance of the vessels. Coincident with these symptoms is another, which continues until approaching devitalization appears. This is the pain excited by heat, and it may be regarded as the second indication of the objective stage, if not concurrent with the first appearance of soreness of the investing membrane.

We have been considering a progressive case. There are many cases where, for some time, there is a kind of balance carried on between these two general conditions. For a time, the objective stage will be most marked, but may decline, when the subjective indications will again appear. Afterwards both may subside and the pulp apparently resume its normal condition. These alternations may repeatedly occur. The distinctiveness of these two series of symptoms are of importance in reference to the treatment to be employed. When the symptoms are subjective, and involve principally the nervous elements of the pulp, or can be kept within those bounds, conservation of the pulp may be attempted with reasonable grounds for success, but whenever the symptoms become objective, and involve the circulating elements of the pulp, the prognosis becomes uncertain, and increasingly speculative as the indications of this condition advance.

*Dr. Darby*—Had listened to the paper with a great deal of interest. It was practical and timely. We all meet with cases where we are sure there is pulp irritation, but find it difficult to define where and what it is. These cases are quite difficult to treat. We do not know how long it is well to let them go on, nor yet can we always decide when it is best to cease external applications, and either remove the filling or drill into the pulp. He related a case in which he felt sure that devitalization would have had to be resorted to, and yet, under local treatment, the pain had unexpectedly ceased, and at present he had no doubt but that the pulp had returned to a normal condition. Dr. Jack's paper was suggestive; he hoped that by following out those observations we might be able to diagnose more clearly than we now can the exact condition of the pulp, so as to apply promptly the treatment best suited to its condition.

*Dr. W. B. Miller*—Exhibited, and, assisted by drawings, illustrated his method of applying the various forms of duplex and other matrices which he has invented. They are practical devices, and will no doubt prove quite useful. In the absence of the drawings it is impossible to give an intelligent idea of their construction, and the manner in which they are applied.

Dr. Sophie E. Feltwell, of Pittsburg, read a paper entitled "Abscess and Its Treatment." The essayist traced the usual course of alveolar abscess, describing its several varieties and noting those points where diagnostic care is needed to avoid mistaking alveolar abscess for allied conditions in the surrounding tissues. The treatment suggested did not differ materially from that usually adopted.

(TO BE CONTINUED.)

